# Microfluidic Production and Characterisation of Cyclosporine A-Loaded Lipid–Chitosan Hybrid Nanoparticles as Candidate Pulmonary Drug Delivery Systems

**DOI:** 10.3390/pharmaceutics18091087

**Published:** 2026-08-28

**Authors:** Pierpaolo Palermo, Davide De Angelis, Elisa Sgarbi, Irene Bassanetti, Michael M. Tunney, Dimitrios A. Lamprou

**Affiliations:** 1 School of Pharmacy, Queen’s University Belfast, 97 Lisburn Road, Belfast BT9 7BL, UKm.tunney@qub.ac.uk (M.M.T.); 2 Chiesi Farmaceutici S.p.A., Largo Francesco Belloli 11/A, 43122 Parma, Italy

**Keywords:** pulmonary disease, microfluidics, solid lipid nanoparticles, solid hybrid nanoparticles, cyclosporine A, peptide delivery

## Abstract

**Backgorund/Objectives:** Respiratory diseases represent a substantial global health burden and require effective localised pulmonary delivery strategies, particularly for poorly water-soluble therapeutic molecules. Nanoparticle-based drug delivery systems, especially those manufactured using microfluidics, have emerged as promising approaches to overcome pulmonary barriers, enhance local drug retention, and reduce systemic side effects. Among these nanocarriers, solid lipid nanoparticles (SLNs) and solid hybrid nanoparticles (SHNs) combine biocompatibility with controlled release and improved formulation stability. **Methods:** In this study, SLNs and lipid–chitosan SHNs were developed using microfluidic technology as candidate platforms for pulmonary drug delivery, with Cyclosporine A (CyA) used as a model hydrophobic cyclic peptide. Nanocarriers were produced using 1,2-dipalmitoyl-sn-glycero-3-phosphocholine (DPPC) and cholesterol as lipids, with low-molecular-weight chitosan incorporated to obtain hybrid systems. Physicochemical properties were evaluated using dynamic light scattering (DLS) and ζ potential measurements, while morphology and structural organisation were investigated using transmission electron microscopy (TEM), Fourier-transform infrared spectroscopy (FTIR), thermogravimetric analysis (TGA), and differential scanning calorimetry (DSC). **Results:** The microfluidic approach enabled the production of nanoparticles with controlled sizes below 200 nm, narrow size distributions, and good reproducibility. In addition, the SHNs exhibited a positive surface charge, high encapsulation efficiency (~80%), and good colloidal and thermal stability. In vitro release studies showed an initial burst release followed by sustained CyA release, reaching approximately 94% cumulative release within 6 h. The Korsmeyer–Peppas model was used as the standard kinetic model. No blank nanoparticles were used as controls in the EE and release assay. **Conclusions:** Overall, these findings support further investigation of microfluidic-produced lipid and hybrid nanoparticles as candidate platforms for pulmonary drug delivery.

## 1. Introduction

Respiratory diseases represent a growing global health challenge and are among the leading causes of morbidity and mortality worldwide [1]. Their development is driven by a combination of environmental exposures, including pollutants and infectious agents, as well as genetic and lifestyle factors such as smoking. Respiratory diseases are associated with persistent symptoms that result in both direct and indirect healthcare costs. In addition, they affect all countries, but with a higher incidence in underdeveloped or developing countries due to the scarcity of resources, both financially and in terms of prevention [2].

It has been estimated that, in sub-Saharan Africa, the incidence of asthma is between 10% and 15% in all age groups, while the prevalence of Chronic Obstructive Pulmonary Disease (COPD) is between 1.7% and 24%. Moreover, disease prevalence is likely to be under-estimated in this region; for example, in Nigeria, there is an under-diagnosis rate of approximately 50% for asthma and 99% for COPD. In Uganda, preventable asthma deaths occur three times more frequently than in high-income countries [3]. Asian low- and middle-income countries demonstrate the highest COPD prevalence and mortality rates, driven by high tobacco consumption in the male population and exposure to household and ambient air pollution for women and children. The incidence of COPD in Central Europe is primarily associated with tobacco and occupational exposures. Mortality rates in these regions substantially exceed those in North America and Western Europe [4].

The treatment of lung diseases is remarkably difficult because of the complex structure of the lungs and the presence of biological barriers. A solution to this issue comes from using drug-loaded nanoparticles (NPs), especially if produced using microfluidic (MF) technologies. Unlike classical drug delivery systems (DDSs), NPs not only overcome biological barriers at the lung level, but are also biodegradable, biocompatible, protect the drug from enzymatic degradation and immune cell attack, and improve pharmacology by increasing therapeutic effect, decreasing toxicity, and allowing controlled and targeted release of the molecule [5].

Among nanoscale carriers, lipid-based systems have garnered considerable interest because of their compatibility, ability to encapsulate both hydrophilic and hydrophobic drugs, and potential for controlled release. Solid lipid nanoparticles (SLNs) are characterised by a solid lipid core that confers structural stability and prolonged drug release profiles compared to more fluid lipid nanoparticles. Recent reviews have highlighted the opportunities and challenges of SLNs as pulmonary carriers, emphasising their physicochemical stability and capacity to deliver complex therapeutic payloads, including peptides and proteins, directly to the lungs [6]. Solid hybrid nanoparticles (SHNs) represent an evolution of traditional lipid nanocarriers, integrating polymers and lipid matrices to synergistically enhance their stability, drug loading, and functional performance. Chitosan has been extensively investigated as a coating and structural component due to its biocompatibility, positive surface charge, and mucoadhesive properties, which may enhance interaction with negatively charged pulmonary epithelium and mucus layers [7]. It has been demonstrated that polymer–lipid hybrid systems can improve drug stability, reduce burst release and modulate pharmacokinetics compared to purely lipid or polymeric systems, highlighting their potential for complex delivery applications [8] of both hydrophobic and hydrophilic APIs.

Despite the promise of lipid and hybrid nanocarriers, conventional formulation techniques, such as thin-film hydration or high-pressure homogenisation, often suffer from limitations in terms of reproducibility, scalability, and tight control over particle size and composition. MF approaches have emerged as robust alternatives, offering precise control over mixing dynamics, rapid solvent exchange, and scalable manufacturing with minimal batch variability. MF methods have been successfully employed to produce lipid nanoparticles with narrow size distributions suitable for inhalation and systemic applications [9]. Although microfluidic fabrication has been extensively explored for classical lipid systems, its application to hybrid compounds combining both polymers and lipids remains underexplored, particularly for pulmonary drug delivery. This gap is, in part, attributable to practical challenges that emerge when processing polymer-containing formulations in MF devices. High polymer concentrations can cause channel clogging due to aggregation of NP precursors on channel walls, which can lead to device failure [10].

In this study, we used a model peptide, Cyclosporine A (CyA), as a drug to be encapsulated inside nanocarriers. CyA is a potent immunomodulatory peptide with demonstrated therapeutic potential in inflammatory and autoimmune lung conditions. However, its clinical utility is often limited not only by poor aqueous solubility and a narrow therapeutic window, but also because it may cause nephrotoxicity and a certain level of susceptibility to opportunistic infections [11]. Therefore, nanoparticle formulations have been explored to improve CyA bioavailability and reduce systemic toxicity [12], but formulating CyA for inhalation remains challenging due to its physicochemical properties. These limitations have motivated extensive research into nanoparticle-based formulations capable of improving CyA solubility, encapsulation and controlled release.

This study describes the development and comprehensive characterisation of microfluidic-produced lipid and lipid–chitosan hybrid nanoparticles for CyA delivery, addressing key design considerations for potential pulmonary applications and investigating the potential of MF as a sustainable and highly reproducible approach for producing personalised nanomedicines that are finely controlled based on all their physicochemical features. By optimising the microfluidic parameters and material composition, nanoparticles with a controlled size below 200 nm, narrow polydispersity, positive surface charge, high encapsulation efficiency, and sustained release profile compatible with further respiratory delivery investigation were achieved. The physicochemical stability of these systems was evaluated under different storage conditions, and their structural organisation was investigated via spectroscopic and thermal analyses.

## 2. Study Rationale

**Figure 1:**
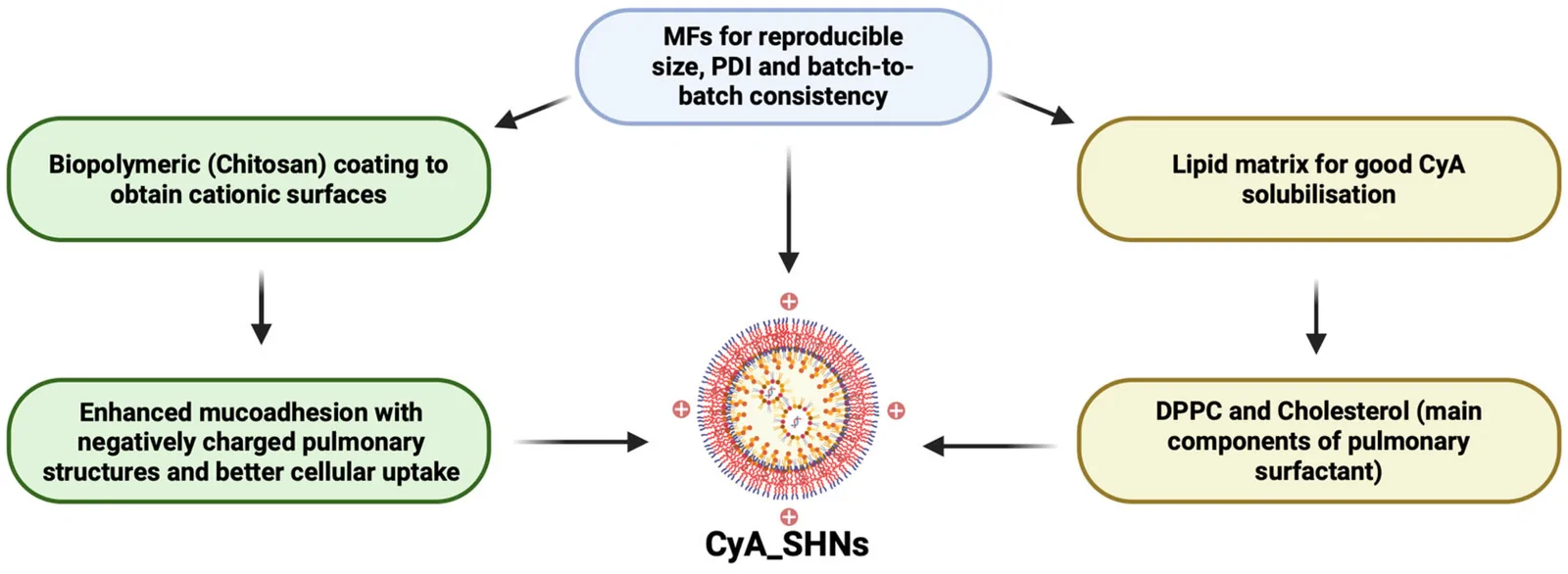
Schematic representation of the MF-based strategy to produce Cyclosporine A-loaded solid hybrid nanoparticles.

An explanatory graphical flow of the formulation strategy is shown in Figure 1. To comprehensively characterise the developed nanoparticles, a series of physicochemical analyses were performed. DLS and ζ potential measurements were used to evaluate particle size, polydispersity index (PDI), and surface charge, providing evidence of chitosan surface coating and preliminary indications of colloidal stability. TEM analysis was carried out to examine particle morphology and assess whether the images were consistent with the proposed lipid–chitosan hybrid architecture. FTIR spectroscopy, TGA, and DSC were employed to investigate drug incorporation, assess potential drug–excipient interactions, and characterise the solid-state and thermal behaviour of the nanoparticles. Storage stability was evaluated to determine the robustness of the formulations under defined storage conditions. Finally, an in vitro drug release study was performed using a dialysis membrane approach to obtain a preliminary release profile and provide initial insight into the release kinetics of the encapsulated drug.

## 3. Materials and Methods

### 3.1. Materials

**Figure 2:**
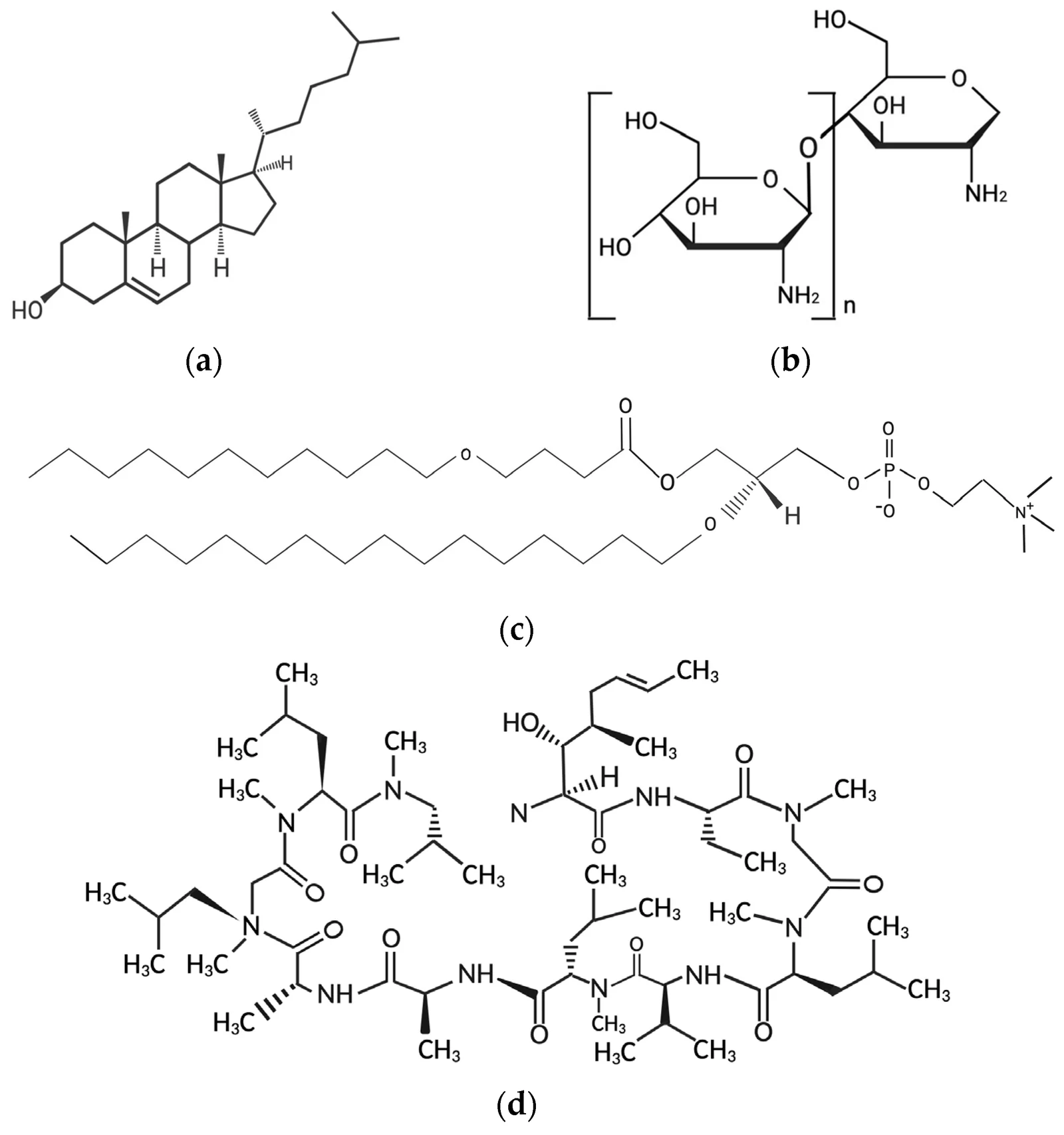
Chemical structures of (**a**) cholesterol, (**b**) LMW chitosan, (**c**) 1,2-Dipalmitoyl-sn-glycero-3-phosphocholine (DPPC), and (**d**) CyA.

Cholesterol (MW: 386.65 g/mol, Sigma grade, ≥99% purity), low-molecular-weight (LMW) chitosan (standard solubility: 1 g/100 mL in 1% acetic acid (*v*/*v*) solution), 1,2-Dipalmitoyl-sn-glycero-3-phosphocholine (DPPC; MW: 734.039 g/mol, 99% purity), Cyclosporine A (CyA, MW: 1202.61 g/mol; used as model peptide), ethanol (purity ≥ 99.8%, HPLC grade), and acetic acid (glacial, purity ≥ 99.9%) were acquired from Merck (Gillingham, UK). The chemical structures of the compounds are shown in Figure 2.

### 3.2. Methods

#### 3.2.1. Microfluidic Chip Design and Manufacturing

The Y-shaped microfluidic chip used in this study was a device previously developed by our group [13] and was created using open-source computer-aided design (CAD) software (TinkerCAD 1.4.x, Autodesk Inc., San Francisco, CA, USA). The design was converted into an STL file and uploaded to Asiga Composer software (version 1.2; Asiga, Alexandria, Australia). The design was positioned at a 20° angle and supported appropriately to optimise printing quality. The chip was printed using an Asiga MAX UV 3D printer at a resolution of 0.0025 mm using PlasCLEAR resin (Asiga, Alexandria, Australia). The internal dimensions of the MF chip were 0.8 × 1.94 × 1.22 mm. To achieve a homogenous mixing between the aqueous and organic phases while operating under laminar flow conditions, the device geometry was designed to achieve a certain degree of chaotic advection. Chaotic advection can be induced by introducing geometric features in the chip design. These elements can repeatedly stretch and reorient the fluid streamlines as the phases flow through the channels, promoting rapid and efficient mixing. This strategy was therefore selected over a straight-channel design, as it allows faster and more efficient mixing, as well as more controlled nanoprecipitation, avoiding phenomena that could compromise nanoparticle homogeneity [13]. Once printing was complete, the device was cleaned, post-processed, and prepared for use in microfluidic applications.

#### 3.2.2. Preparation of NPs

**Figure 3:**
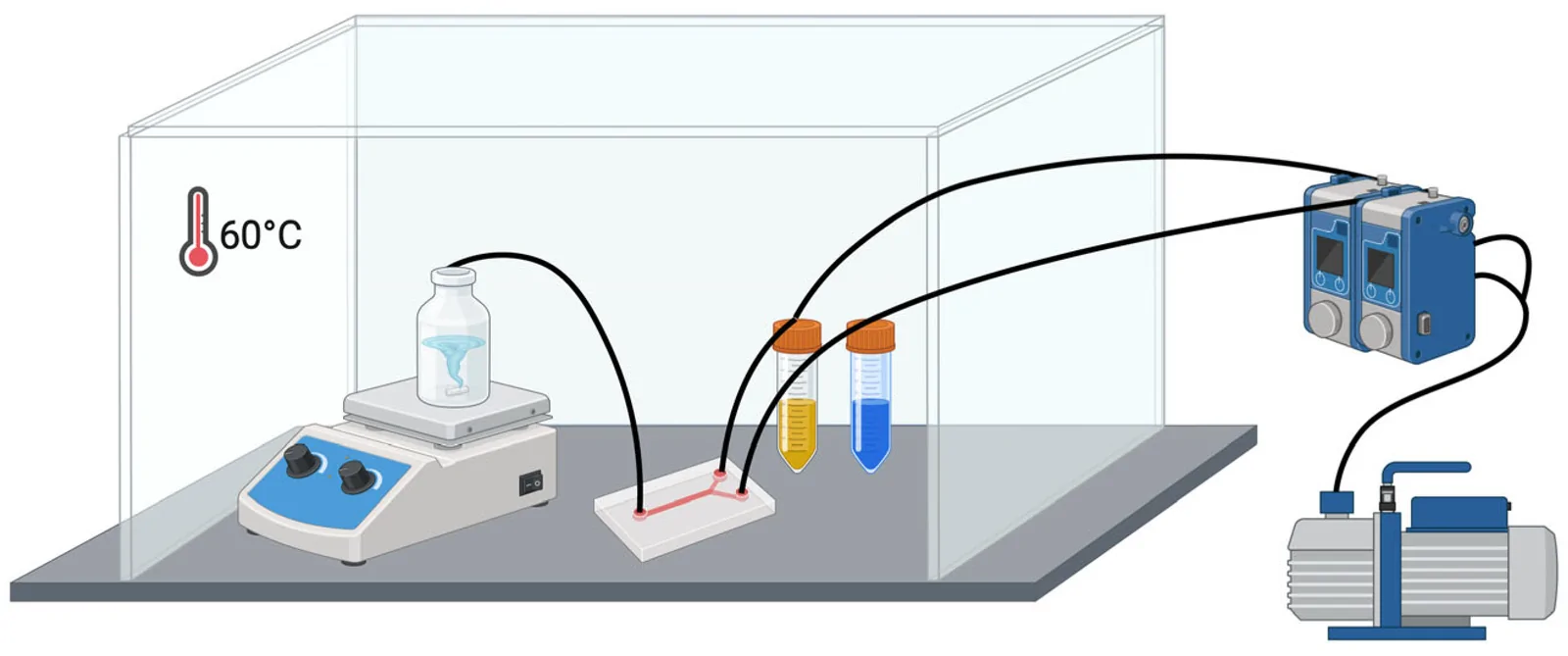
Representative image of the microfluidic “in-house” system set-up for empty and loaded NP production in a controlled-temperature environment.

For the microfluidic production of lipid and hybrid nanoparticles, a Fluigent (Paris, France) Lineup Flow EZTM was used. To ensure the complete dissolution of all materials, the microfluidic environment was set to 60 °C using an “in-house” system first introduced by this group in a previous study [14] (Figure 3). Briefly, for empty SLNs, an ethanol stock solution composed of DPPC and cholesterol in pre-selected mass ratios was prepared to reach [1 mg/mL] using 70% *w*/*w* DPPC and 30% *w*/*w* cholesterol, while the aqueous phase was filtered using Milli-Q H_2_O. The two phases were inserted in the two microfluidic chambers and injected into the MF chip, using a range of total flow rates (TFRs) from 0.5 to 4 mL/min and a flow rate ratio (FRR) of 1:2 (lip:aq), as reported previously [15]. All investigated variables are reported in Table 1, with the designations F1–F4 referring to the different TFRs used. Regarding SHNs, a hybrid feature was achieved by adding low-molecular-weight chitosan solubilised in an aqueous phase enriched with 1% acetic acid (*v*/*v*) [16]. The most suitable TFRs and FRRs obtained from the empty SLN preparation were then used for the subsequent formulation of SHNs, in which the lipid concentration remained the same as that used for SLNs, whilst the chitosan concentration in the aqueous phase was [0.5 mg/mL], so that the polymer-to-lipid ratio was 1:2. Microfluidic manufacturing was carried out for 1 min, collecting a total sample of 2 mL for all the investigated SLN and SHN combinations, following a 2:1 (aq:lip) ratio. Excess ethanol was evaporated using constant vortex stirring for approximately 1 h.

For CyA-loaded SHNs, the model cyclic peptide was solubilised in the ethanolic phase at a concentration of approximately [0.30 mg/mL], corresponding to a 1:3.33 drug-to-lipid ratio, and mixed using a magnetic stirrer until complete solubilisation was achieved. Microfluidic procedures were carried out for 1 min, and 2 mL samples were collected using a 2:1 aqueous-to-lipid phase ratio. Excess ethanol was evaporated under constant vortex stirring for approximately 1 h.

#### 3.2.3. Physicochemical Characterisation

##### Dynamic Light Scattering and ζ Potential

Dynamic light scattering (DLS9 system NanoBrook Omni particle sizer; Brookhaven Instruments, Holtsville, NY, USA) was used to measure the NP particle size, polydispersity index (PDI), and ζ potential. Briefly, 100 μL of NP formulation was diluted with 2.4 mL of filtered Milli-Q H_2_O and poured inside a glass cuvette. For the following ζ potential analyses, 1.8 mL from the previous dilution was analysed using a BI-SREL electrode from NanoBrook. Each sample was run in triplicate. All results are expressed as means ± standard deviations.

##### Stability Studies

The particle size, PDI, and ζ potential were measured for a total period of 28 days, beginning with the recording on day 0 of SHN preparation, followed by days 1, 2, 7, 14, 21, and 28. All samples were kept under constant storage conditions at three different temperatures: 4 °C, 25 °C, and 37 °C. Specifically, 4 °C was used to evaluate stability preservation and degradation control, 25 °C to mimic the formulation’s behaviour at room temperature (RT), and 37 °C to address the behaviour at in vivo temperatures.

##### Transmission Electron Microscopy (TEM)

By adopting a projection of elastically scattered electrons, TEM was employed to investigate the morphology and surface topography of loaded SHNs at the micro-/nanoscale level. For sample preparation, a small aliquot of the SHN suspension was deposited onto formvar/carbon-coated copper grids and left to dry for 20 min in a fume hood. SHNs were then negatively stained with 2% (*w*/*v*) aqueous uranyl acetate solution. Excessive staining solution was subsequently removed, and the grids were left to dry for about 2 to 3 min, prior to analysis. Images were obtained using a JEOL JEM-1400 Plus Transmission Electron Microscope (JEOL Ltd., Tokyo, Japan) with a 120 kV voltage.

##### Fourier-Transform Infrared Spectroscopy (FTIR)

FTIR analyses were performed on individual raw materials in powder form and on the selected SHN formulations as liquid dispersions to evaluate the structural organisation and self-assembly behaviour of the systems, excluding any undesired chemical interactions among the formulation components. Spectra were acquired using an attenuated total reflection Fourier-transform infrared (ATR-FTIR) spectrometer (Nicolet iS50 FTIR; Thermo Fisher Scientific, Waltham, MA, USA) with a built-in ATR accessory. Each sample was analysed in triplicate under an inert atmosphere in the spectral range of 4000–400 cm^−1^, collecting 32 scans at a resolution of 4 cm^−1^, with a data interval of 1 cm^−1^. Background absorption was subtracted from each scan.

##### Thermogravimetric Analysis (TGA)

TGA was used to evaluate the thermal behaviour of the raw materials and formulations. TGA was performed using a Thermal Advantage Model Q500 thermogravimetric analyser (TA Instruments, New Castle, DE, USA) and standard aluminium pans. Prior to TGA analysis, the SHN formulations were centrifuged at 14.800 rpm for 30 min at 4 °C using the Centrifuge 5425 R (Eppendorf UK Ltd., Stevenage, UK) to remove the supernatant. The pellets were dried and then analysed using TGA.

Each analysis was performed under a dynamic heating protocol ranging from 20 to 80 °C at a heating rate of 10 °C/min. A temperature of 80 °C was used as the final temperature to evaluate raw-material and formulation stability in the in-house MF system experimental conditions.

##### Differential Scanning Calorimetry (DSC)

DSC was performed using a DSC Q200 from TA Instruments (TA Instruments, New Castle, DE, USA) using closed aluminium pans. SHN suspensions were centrifuged at 14.800 rpm for 30 min at 4 °C, the supernatant was removed, and the remaining sample was left to dry prior to analysis.

DSC analyses were performed up to 80 °C, following the same workflow used for TGA analyses and adopting a heating rate of 10 °C/min.

#### 3.2.4. Encapsulation Efficiency (EE) and In Vitro Drug Release Profile

A stock solution of CyA was prepared at a concentration of 1.5 mg in 10 mL of ethanol, yielding a stock concentration of 150 μg/mL. A working solution was obtained by diluting the stock in Phosphate-Buffered Saline (PBS) using a 4:1 (EtOH:PBS) ratio, resulting in a final concentration of 120 μg/mL. Serial dilutions were then prepared using standards (STDs): PBS (4:1, *v*/*v*) dilutions to generate a calibration curve ranging from 100 to 0.3 μg/mL. The detection wavelength was 210 nm. Due to extremely noisy wavelengths, aliquots of EtOH:PBS with the same serial dilutions as the STDs were measured alongside the STDs. The calibration curve showed excellent linearity over the tested range, with a coefficient of determination, R2 = 0.9969. For the encapsulation efficiency evaluation, SHNs were centrifuged at 14.800 rpm for 30 min at 4 °C, after which the supernatant was removed and kept for EE measurement. The remaining pellet was washed using 1 mL of fresh PBS, centrifuged again to extract any drug that could have been adsorbed on the SHN surface and kept for EE analysis. EE% was calculated using Equation (1).

(1) $$EE\%=\left(\frac{Total\;mass\;of\;API\;added\;to\;the\;formulation\;-\;Total\;mass\;of\;the\;unencapsulated\;API}{Total\;mass\;of\;API\;added\;to\;the\;formulation}\right)\times 100$$

Dialysis tubing was used for drug release studies, which were performed in triplicate. Dialysis bags (cellulose membrane, average flat width 10 mm, 0.4 in.; MWCO 14,000, Sigma Aldrich, Gillingham, UK) were sterilised by boiling in deionised water and then conditioned in PBS, which was also used as the release medium, for 48 h. Briefly, after 1 mL of SHN formulation was centrifuged at 14,800 rpm for 30 min, 500 μL of suspension was removed and replaced with 500 μL of PBS before transfer into the dialysis bags. A dilution factor of 2, as used in other studies with hydrophobic APIs [17], was applied to minimise precipitation or degradation associated with the hydrophobic nature of CyA. The dialysis bags were then transferred into 15 mL centrifuge tubes filled with PBS and placed in a temperature- and agitation-controlled shaking water bath (OLS Aqua Pro Shaking Water Bath, Grant Instruments, Royston, UK) at 37 °C and 100 rpm. Measurements were taken from the external medium by withdrawing 1 mL aliquots and replacing them with 1 mL of fresh PBS to support sink conditions. Measurements were performed at 30 min, 1 h, 2 h, 3 h, 4 h, 5 h, and 6 h. The drug release profile was evaluated using a Multiskan SkyHigh Microplate Spectrophotometer UV/Vis (Thermo Fisher Scientific, Waltham, MA, USA) at 210 nm with a Q6 quartz cuvette. Aliquots of PBS and ethanol were withdrawn at the same timepoints as the CyA formulation, and their absorbance was subtracted from that of the samples. No blank nanoparticles were used as controls; therefore, this should be considered a limitation of the release study, and blank SHNs should be included in future work to account for possible absorbance contributions from excipients or nanoparticle components.

#### 3.2.5. Statistical Analysis

All experiments were performed in triplicate, where appropriate, and data are reported as means ± standard deviations. Data calculations were performed using Microsoft Excel. Statistical comparisons were performed using GraphPad Prism 9. Two-way ANOVA was used where two independent variables were assessed, followed by Tukey post hoc multiple-comparison tests when applicable. Each sample was measured three times, using samples produced in triplicate (*n* = 9). Differences were considered statistically significant at * *p* ≤ 0.0332, ** *p* ≤ 0.0021, *** *p* ≤ 0.0002, and **** *p* ≤ 0.0001. Specific comparisons and significance levels are indicated in the corresponding figure legends where statistical testing is reported.

## 4. Results and Discussion

### 4.1. Dynamic Light Scattering (DLS)

This study investigated the production of empty lipid SLNs with dimensions ≤ 200 nm for efficient resistance to mucoadhesion and penetration of airway mucus [18], PDI ≤ 0.25, and negative ζ potential because of the negatively charged DPPC and cholesterol. To optimise the production method, different TFRs were screened to investigate their influence on the SLN characteristics.

**Figure 4:**
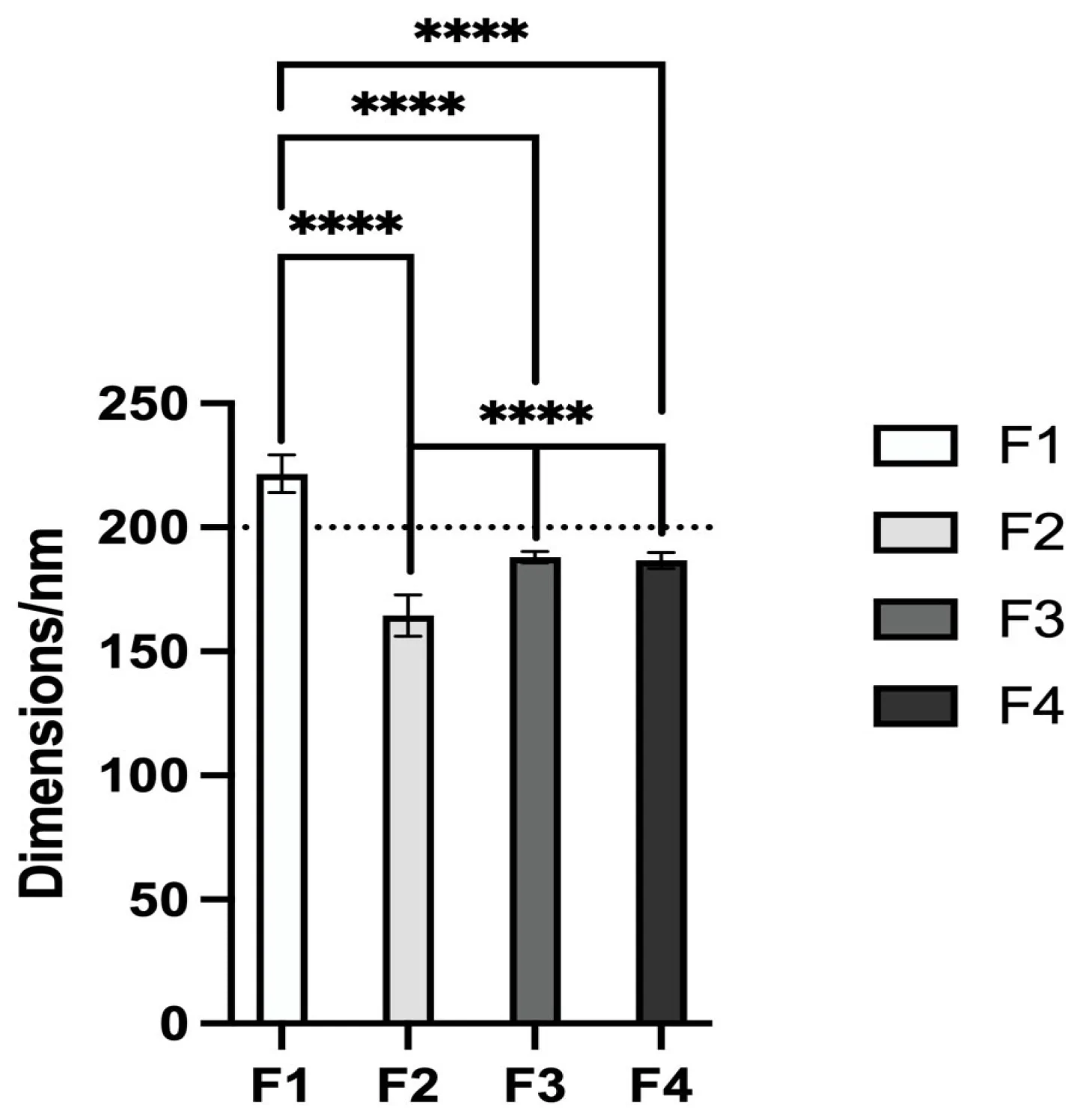
Diameters (nm) of empty lipid nanoparticle formulations (F1–F4). Data are expressed as means ± standard deviations. All size measurements were taken on day 0 of formulation, and samples were analysed in triplicate. Dotted line represents target diameter threshold (200 nm). Statistical significance was assessed by Two-way ANOVA, followed by Tukey’s post-hoc test (**** *p* ≤ 0.0001).

Figure 4 shows the mean diameters of the four formulations (F1–F4), expressed in nanometres. F1 exhibited the largest particle size, with an average diameter of approximately 220 nm, suggesting less efficient size control or reduced packing during nanoparticle formation. In contrast, F2 showed a smaller size of approximately 165 nm, indicating improved nanoparticle compaction owing to a more favourable TFR of 1 mL/min. Formulations F3 and F4 presented comparable particle dimensions, both close to 185–190 nm, suggesting no compositional differences under the investigated preparation conditions. Compared with the other formulations, the ethanol fraction in F4 led to significant solvent evaporation during the stirring process, resulting in an uncontrolled shift in the final formulation composition and compromising batch-to-batch reproducibility. According to the evaporation dynamics of binary mixtures, when ethanol–water droplets are formed, an uncontrolled flash evaporation of the most volatile component can be inducted during collection and early stirring stages [19]. Conversely, F3 prevents premature solvent evaporation and is considered better because it is a more environmentally sustainable choice. The relatively small standard deviations indicate good reproducibility and narrow size distributions for all formulations. Overall, except for F1, which was slightly larger than 200 nm, the other formulations showed dimensions < 200 nm, a size range that may be relevant for the further development of pulmonary nanocarrier systems.

As shown in Table 2, all formulations exhibited PDI values below 0.25, indicating narrow particle size distributions and good formulation homogeneity. Among them, F3 and F4 exhibited particularly low PDI values (0.095 ± 0.041 and 0.057 ± 0.043, respectively), suggesting a highly uniform nanoparticle population. The low PDI values observed for all formulations confirmed the effectiveness of the microfluidic preparation method in producing well-controlled SLNs. In addition, the remarkably low PDI of F3 and F4 indicates superior colloidal uniformity, which may be attributed to the optimised lipid composition and/or flow conditions during microfluidic assembly. Despite the improvement in mixing homogeneity reflected by the PDI results, ζ potential did not follow the same monotonic trend. In F1-F3, solvent mixing remained sufficiently slow, allowing effective surface charge for SHNs. It has been hypothesized that a TFR of 2 mL/min likely represents a critical transition state. At this flow rate, the precipitation temporarily distorts the orientation of DPPC headgroups, causing a mild negative ζ potential. Using a TFR of 4 mL/min, high shear forces promote structural relaxation and a different packing of cholesterol into the hydrophobic core, partially restoring the native DPPC surface charge configuration. All formulations displayed a mildly negative ζ potential, consistent with the presence of anionic lipid components on the particle surface.

Based on the evidence and results presented above, especially in terms of dimensions and PDI, 2 mL/min TFR and FRR 1:2 (lip:aq) were chosen as the best parameters for both empty and loaded SHNs. The dimensions of newly obtained empty SHNs were in the nanometre range, reaching an average of 156.82 ± 11.35 nm, with a PDI of 0.215 ± 0.023 and a highly positive ζ potential (25.65 ± 4.33 mV). Consequently, there was a notable increase in PDI and a switch in ζ potential from negative to positive values, caused by the introduction of low-molecular-weight chitosan in the acidic aqueous phase [20]. This results in a higher number of positive charges on the outer surface of the SHNs, which may support electrostatic interactions with the negatively charged respiratory epithelium and mucus layer, caused by the high presence of phospholipids, especially DPPC and cholesterol, in the pulmonary surfactant (PS). These interactions may potentially increase residence time and cellular uptake, although this requires confirmation using mucus- and cell-based models.

Accordingly, with empty SLNs and SHNs, the dimensions of loaded SHNs were stable following drug encapsulation, with an average diameter of 160.54 ± 6.50 nm, measured between three replicates (Table 3). PDI and ζ potential were also within the previously investigated ranges. Notably, following the encapsulation of CyA into SHNs, there was a decrease in ζ potential, from an extremely positive value of 25.65 ± 4.33 mV to a lower but still positive value of 16.13 ± 1.15 mV.

### 4.2. Stability Studies

**Figure 5:**
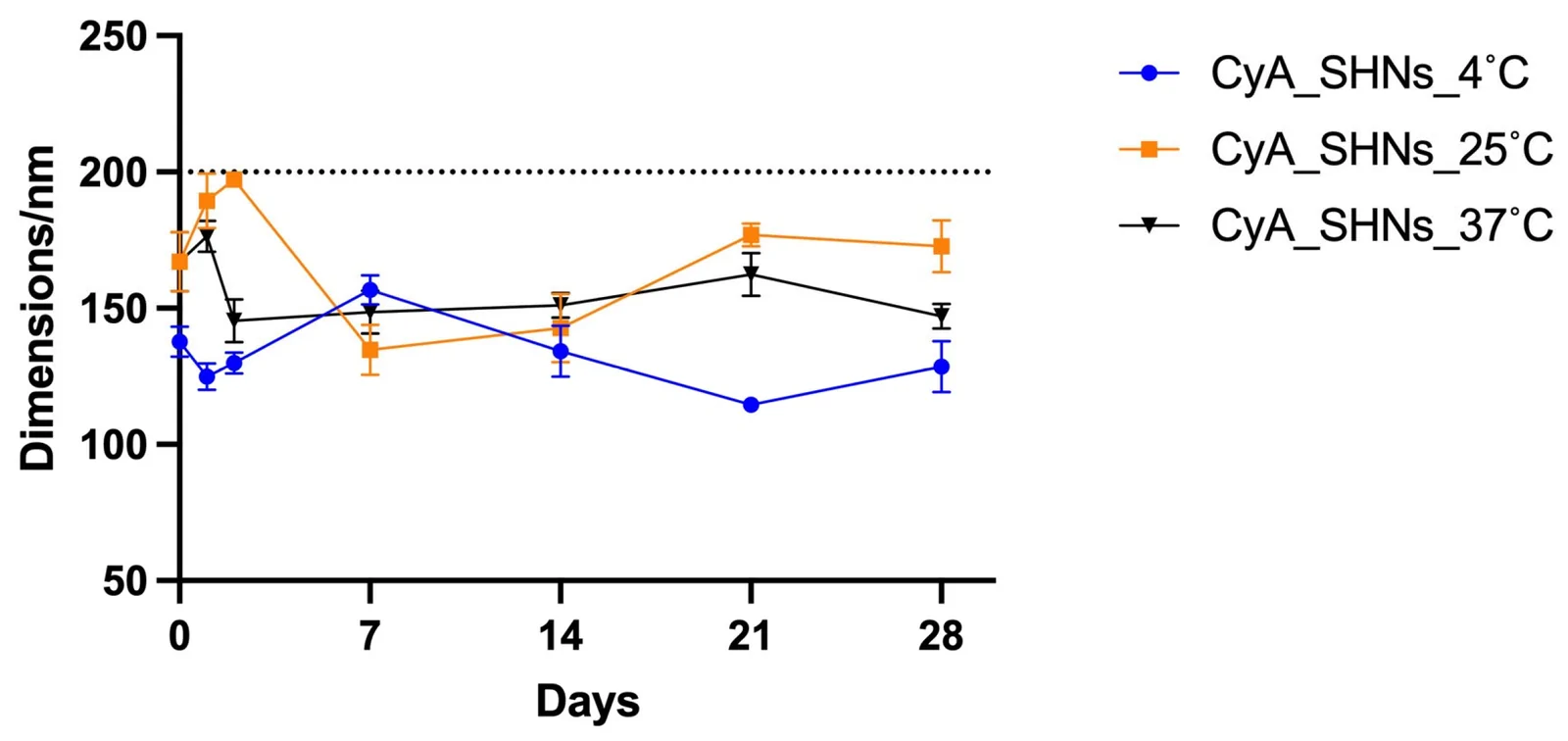
Particle size monitoring conducted on SHN formulations for a period of 28 days, using 4/25/37 °C as storage conditions. Dotted line represents target diameter threshold (200 nm).

Stability studies of the SHN formulations showed good and promising physical stability. As can be seen in Figure 5, all formulations maintained particle sizes below 200 nm throughout the study, as indicated by the reference line. The fluctuating stability observed at 4 °C can be attributed to lipid-phase transitions and surface rearrangements, leading to aggregates and greater size variability over the investigated period. In addition, the formulation stored at 4 °C showed a tendency to form aggregates, which disappeared when the formulation was left at room temperature for approximately 1 h prior to DLS analysis. Storage at room temperature (25 °C) resulted in a higher increase in particle size, likely due to partial lipid reorganisation and increased Brownian motion. At 37 °C, the higher thermal energy may reduce molecular mobility and minimise lipid rearrangement, limiting nanoparticle aggregation and fusion phenomena. Nevertheless, the absence of a pronounced or irreversible increase in size suggests that the investigated SHNs retain acceptable colloidal stability, especially under physiological temperature conditions.

The other two fundamental parameters for the physical analysis of SHNs, PDI and ζ potential, were also monitored for a period of 28 days. PDI and superficial charge values are expressed as averages and are presented in Table 4. Briefly, all formulations showed values close to 0.2 PDI, indicating narrow and homogenous particle size distributions, with minor differences observed among the three storage conditions. This suggests that temperature did not significantly affect the homogeneity of the nanoparticle population over the evaluation period. This indicates a good colloidal stability. The ζ potential values were constantly positive, and even if small variations in surface charge were detected, the ζ potentials ranged from +13.32 to +15.20 mV. Positive values were attributed to the presence of chitosan on the SHN surfaces under all storage conditions, confirming the structural integrity of the coating. In addition, the preservation of surface charge at 37 °C is relevant for further pulmonary delivery investigation, as it may support electrostatic interactions with negatively charged respiratory epithelia and mucus.

Overall, these results demonstrate the ability of loaded SHNs to maintain a positive ζ potential and low PDI values across all storage conditions, indicating the robustness of the formulation and supporting its suitability for biomedical applications.

These findings indicate that all storage conditions can be considered suitable for maintaining colloidal stability over the investigated period. Of particular interest was the finding that 37 °C showed no significant changes from day 2 until day 28, supporting the potential applicability of SHNs in biological and pulmonary delivery settings, pending further respiratory-specific evaluation.

### 4.3. Transmission Electron Microscopy (TEM)

**Figure 6:**
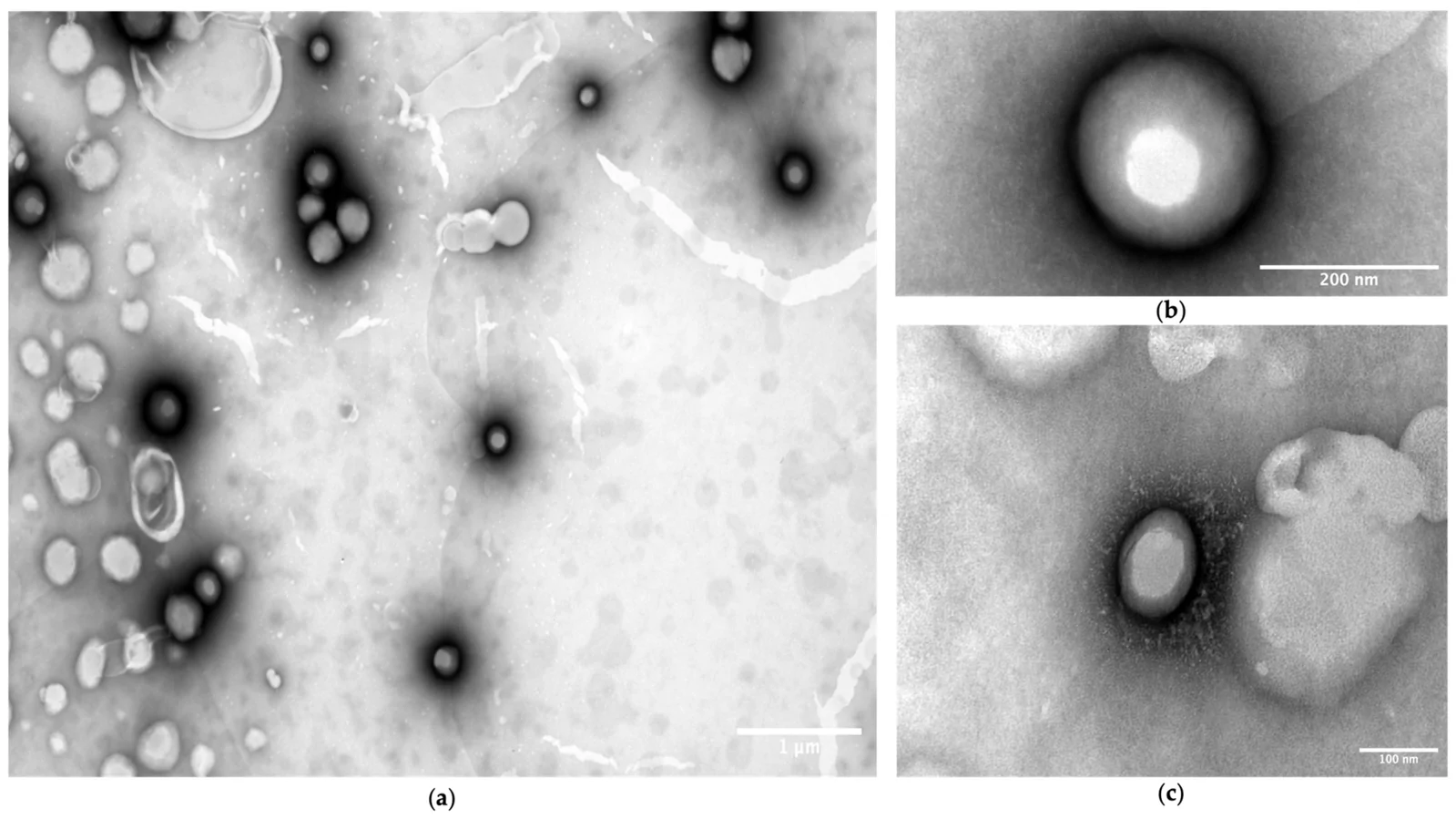
TEM images of SHN formulation showing (**a**) the nanoparticle population (8 K magnification, 1 μm) (**b**) and (**c**) representative individual nanoparticles using 40 K magnification (200 nm) and 80 K magnification (100 nm), respectively. The contrast observed after negative staining is consistent with the proposed lipid–polymer hybrid structure.

The above TEM picture (Figure 6) shows the hybrid lipid–polymer nanoparticles deposited on the grid. In the overview image acquired at 8 K magnification, the field revealed a discrete and well-dispersed nanoparticle population. At higher magnifications of 40 K and 80 K, individual SHNs displayed a spherical morphology with contrast patterns consistent with the proposed lipid–chitosan hybrid structure. The electron-lucent core and darker outer region observed after uranyl acetate staining are consistent with a core–shell organisation, potentially reflecting differential staining affinity between the hydrophobic lipid core and the chitosan outer layer. However, this observation alone cannot be considered conclusive evidence of a defined core–shell architecture. Complementary microscopy techniques would be required to fully define and confirm the structural architecture of the SHN systems. Occasional particle clusters were observed, likely due to the drying and staining processes during sample preparation, which can induce morphological rearrangements [21]. Overall, the TEM images support the formation of nanoscale SHNs with a morphology potentially consistent with the proposed hybrid lipid–chitosan system.

**Figure 7:**
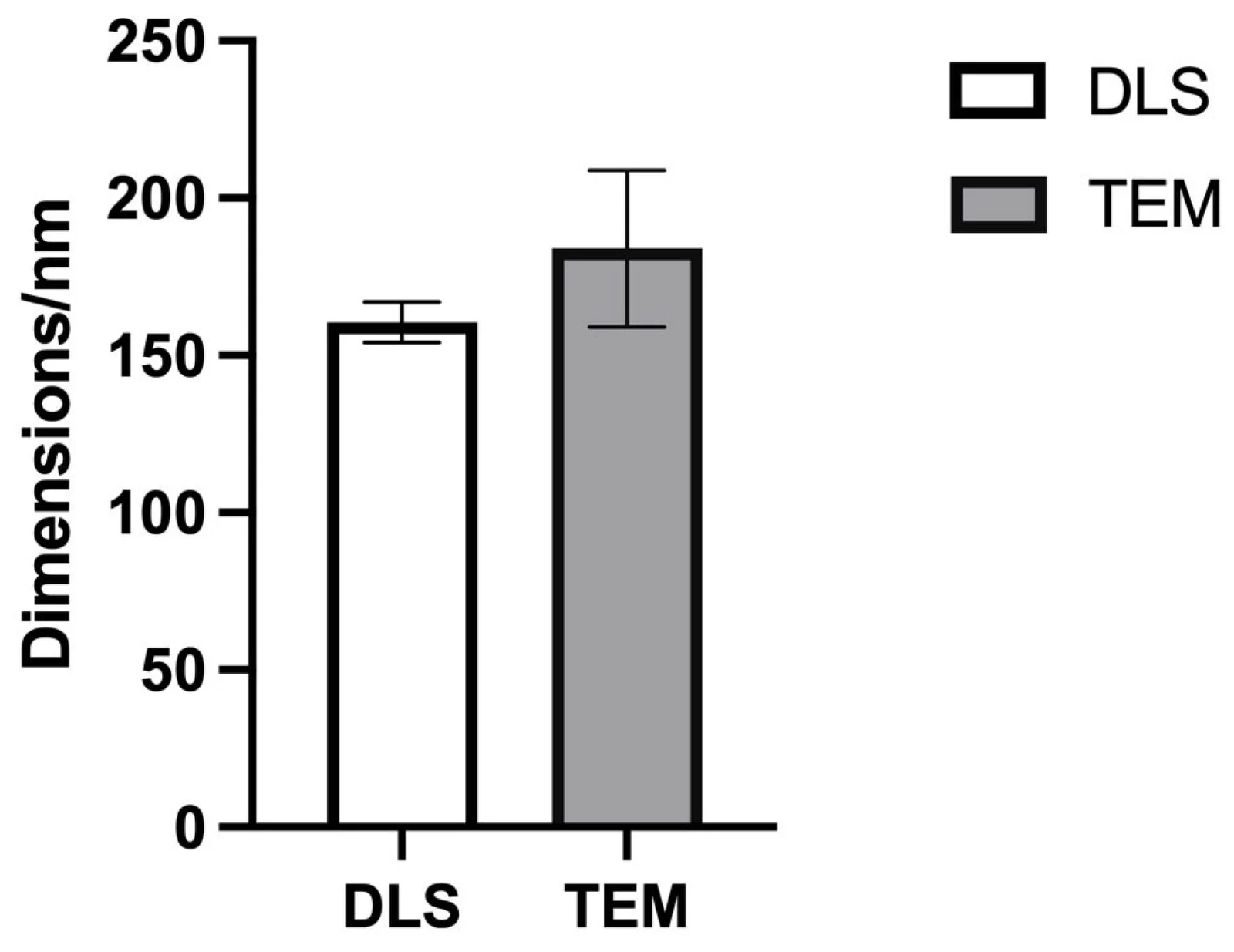
DLS and TEM comparison of SHNs. Data are expressed as means ± standard deviations.

Figure 7 displays a comparison between SHN measurements by DLS and TEM. The TEM-derived mean diameter (183 ± 24.88 nm) is slightly higher than the DLS measurement (160.54 ± 6.5 nm). Larger dimensions observed by TEM could be caused by the sample drying and preparation, during which the hybrid lipid–chitosan shell may undergo flattening or spreading on the grid surface. Nonetheless, even with this variability in dimensions, TEM size distribution remains close to the targeted 200 nm threshold, supporting overall consistency between the two techniques and potential formulation suitability for pulmonary applications.

### 4.4. Fourier-Transform Infrared Spectroscopy (FTIR)

**Figure 8:**
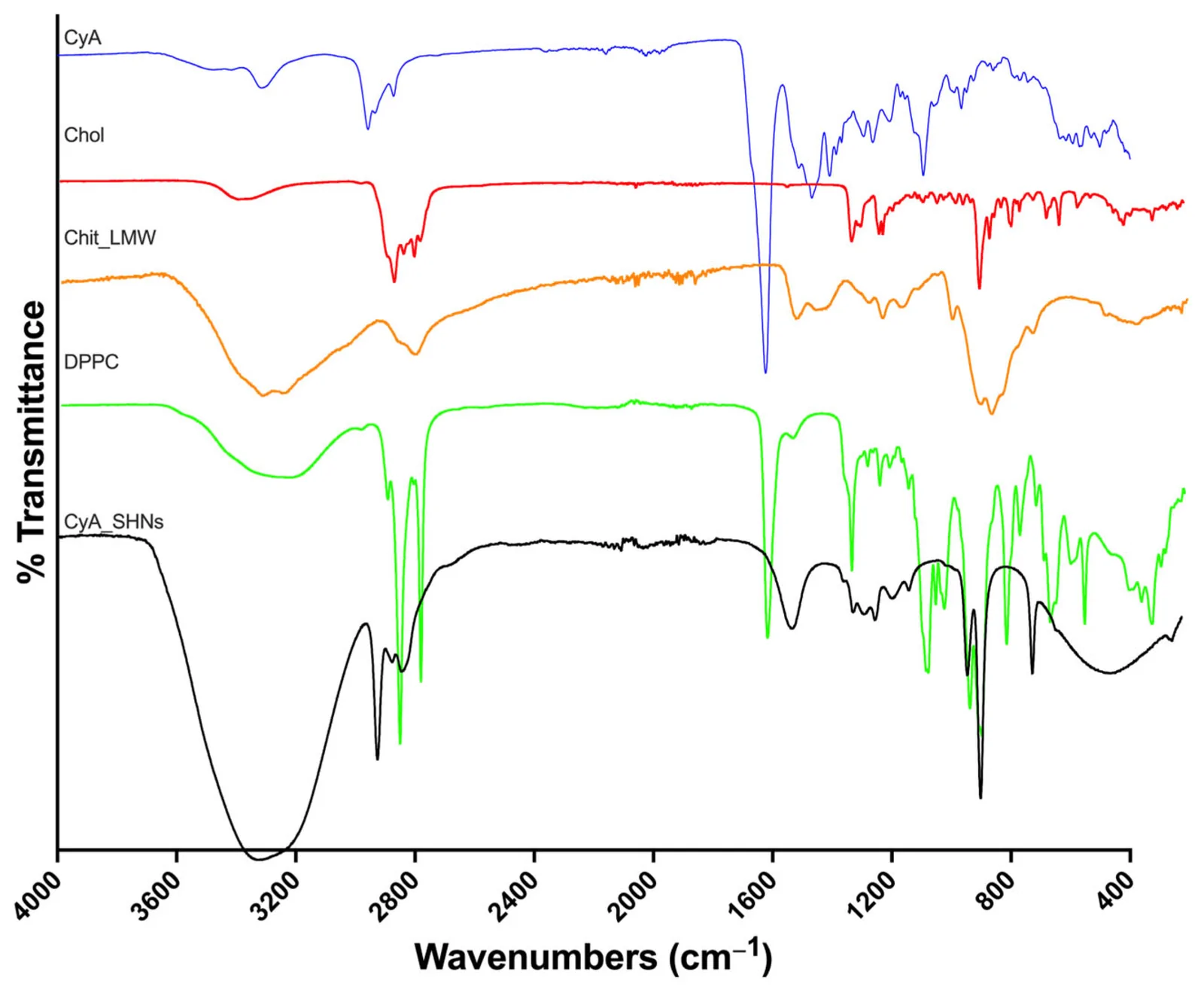
ATR-FTIR spectra of raw materials and loaded SHNs, recorded in the 4000–400 cm^−1^ range.

ATR-FTIR analyses were conducted on the raw materials and the selected SHN formulations. As shown in Figure 8, characteristic absorption bands associated with CyA were identifiable in the loaded formulations, supporting CyA incorporation within the nanoparticulate system.

CyA exhibits a broad absorption band between 2870 and 2960 cm^−1^, attributed to C–H stretching bands, which can also be noted in the formulation profile. Additional characteristic peaks include the C=O stretching vibration at 1623 cm^−1^, which was identified, even though it was slightly shifted and smaller in size, in the SHN spectrum. This may indicate a reorganisation of the native intramolecular hydrogen-bonding pattern of CyA, possibly due to interactions with the surrounding lipids and with -OH/-NH_2_ of the chitosan matrix, potentially suggesting a degree of molecular interaction between the API and the excipients within the nanoparticle formulation. Other notable peaks in the SHNs are the one present at 1094 cm^−1^, attributable to the C–O stretching vibration, which is typical of secondary alcohols [22], and the one present at 966.1 cm^−1^ peak, which may be ascribed to CH-CH trans bonds. However, it should be noted that FTIR results are consistent with CyA incorporation within the SHN matrix but do not, by themselves, provide definitive information on the specific molecular-level interactions occurring between drugs, lipids and chitosan.

The strong bands associated with DPPC (C–H stretching and phosphate vibrations) and cholesterol are preserved in the loaded formulation, confirming that the lipid structure remains intact after drug loading.

In the SHN formulation spectrum, the characteristic CyA bands appeared attenuated and partially overlapped with those of the lipid matrix and chitosan. The reduced intensity and band broadening indicate the physical entrapment of CyA within the lipid core and subsequent hydrogen-bond interactions with the chitosan and phospholipid head groups. Notably, no signs of chemical interactions or degradation phenomena were observed, indicating that there was no free CyA in the crystalline state.

Overall, the FTIR results support the formation of a stable, self-assembled hybrid nanoparticle system in which CyA is successfully incorporated without altering its chemical structure.

### 4.5. Thermogravimetric Analysis (TGA)

**Figure 9:**
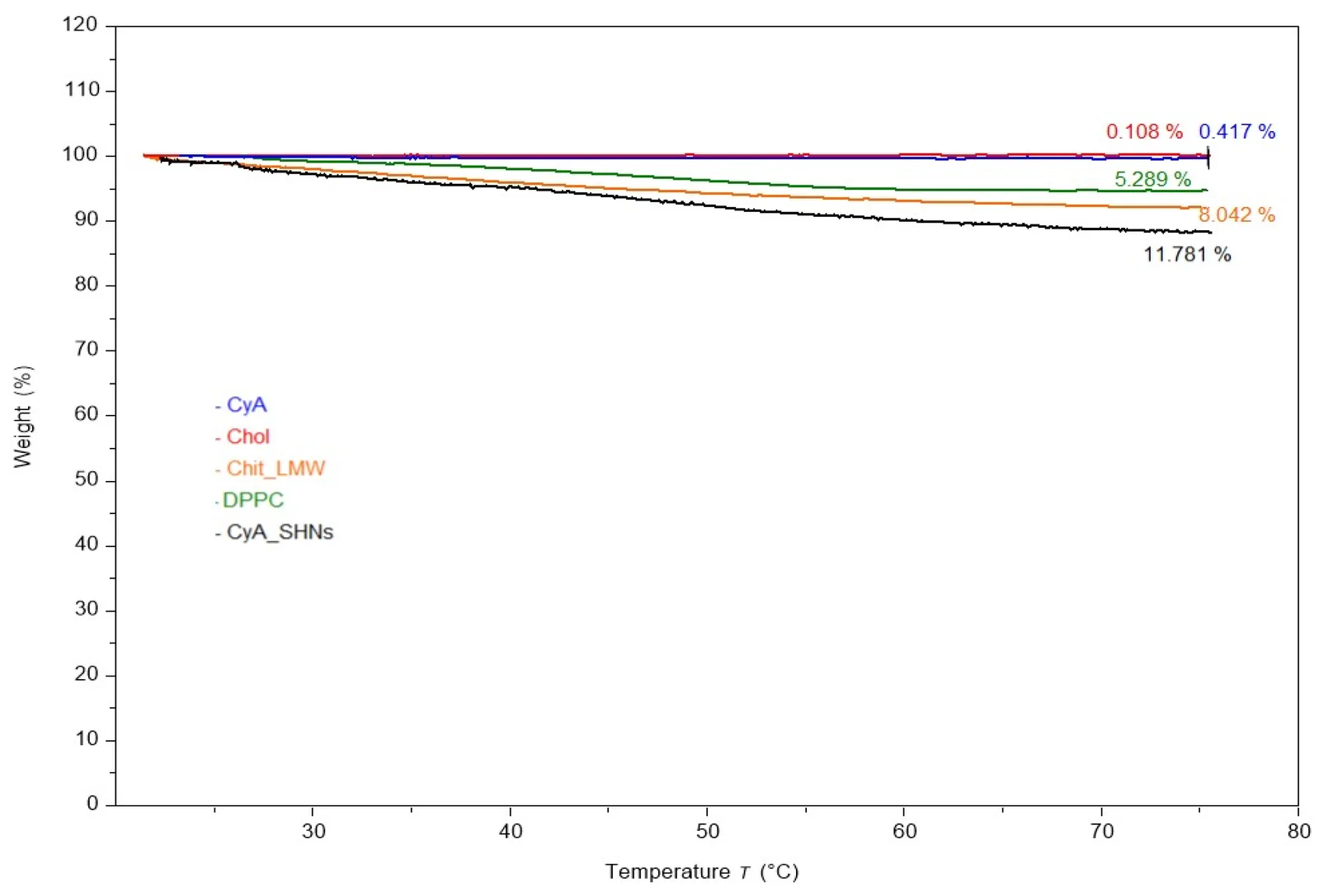
TGA curves of raw materials and SHN formulations showing compound percentage weight loss as a function of temperature. Heating rate used: 10 °C/min; temperature range: 20–80 °C.

TGA analysis, displayed in Figure 9, was used to evaluate the raw materials and SHN formulation stability at 60 °C, the temperature used during the microfluidic manufacturing process. The investigated temperature range was set from 20 °C to 80 °C. All samples exhibited distinct thermal stabilities, reflecting their chemical and physical features.

Low-molecular-weight chitosan shows an initial weight loss at lower temperatures, which can be attributed to the evaporation of physically absorbed and bound water, followed by a gradual mass decrease until 9%, as has been achieved in previous studies [16], associated with polymer chain degradation and depolymerisation of the polysaccharide backbone.

DPPC and cholesterol show different behaviours: cholesterol shows high thermal stability over the investigated temperature range, displaying a usual %weight loss starting around 220–280 °C [23], while DPPC displayed a more pronounced and continuous weight loss until 5% [24], indicating a lower thermal stability in the investigated range.

CyA usually presents a slow decomposition, starting from temperatures close to 240 °C, caused by the dihydroxylation of the drug [22]. CyA showed minimal weight loss in the investigated range until 80 °C.

The observed weight loss in the CyA-loaded SHN pellet primarily reflects evaporation of residual water and solvent from the hybrid nanoparticle system rather than chemical degradation of CyA. In the same temperature range, pure CyA showed minimal weight loss, supporting its thermal stability under these conditions. The larger mass loss observed for the SHN pellet is therefore more likely associated with the hydrated lipid–chitosan matrix and residual solvent content than with API decomposition. Consequently, the TGA data should be interpreted primarily as evidence of formulation behaviour under the processing temperature range rather than definitive proof of enhanced thermal protection of the API. Despite the weight loss beginning before 60 °C, the environmental temperature employed does not affect the SHN formulation process or the obtained structure.

### 4.6. Differential Scanning Calorimetry (DSC)

**Figure 10:**
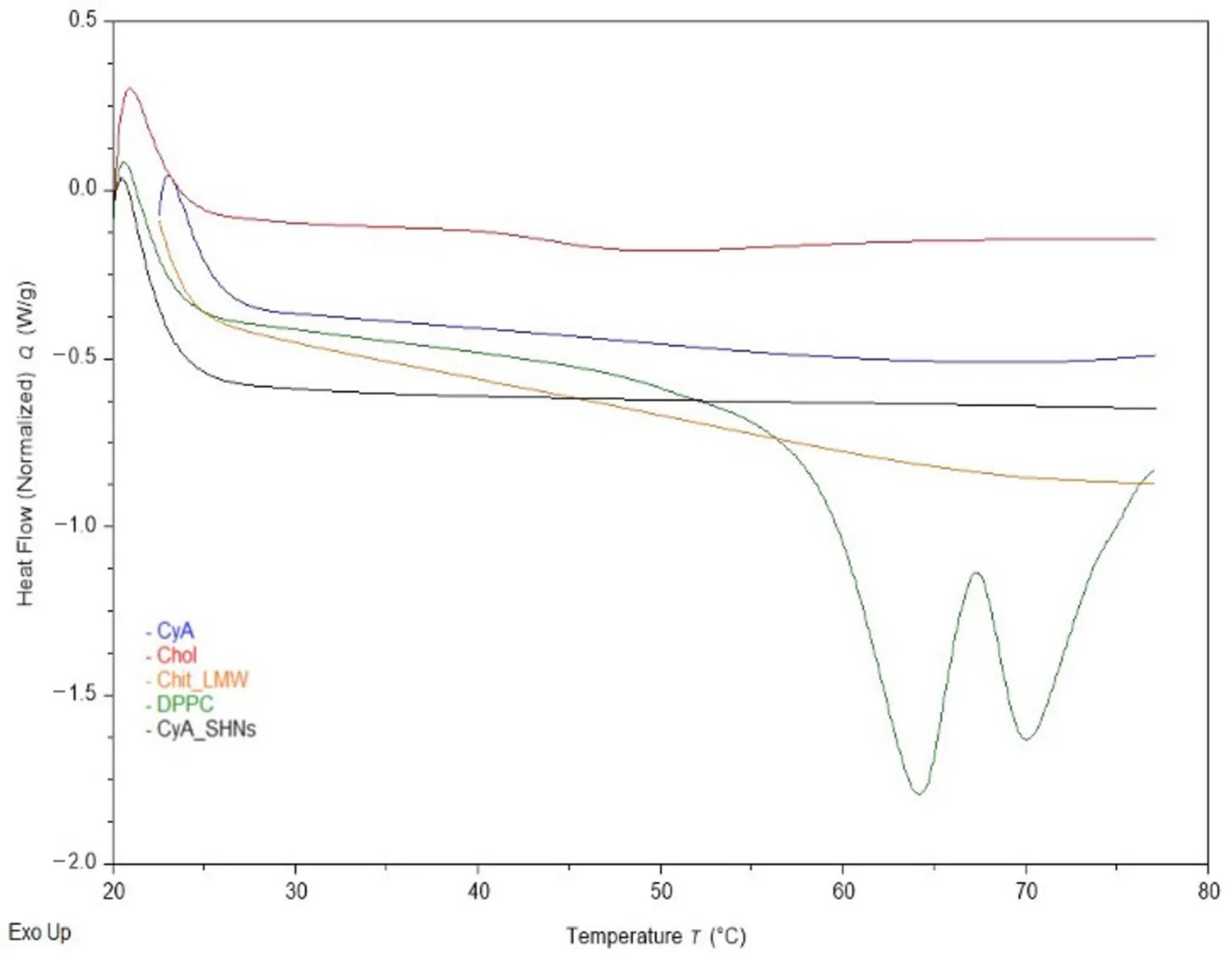
DSC thermograms of raw materials and SHN formulations. Heating rate used: 10 °C/min; temperature range: 20–80 °C.

Differential scanning calorimetry was also performed to further investigate the thermal behaviour of individual components and SHN formulations, with thermograms displayed in Figure 10. Regarding raw materials, chitosan showed a gradually sloping baseline with no clear thermal events in the temperature range. This polymer usually shows an endothermic peak at 100 °C, probably caused by water loss from hydrophilic groups, as well as an endothermic peak above 300 °C after its degradation [25].

The thermogram of DPPC displayed thermal events within the investigated temperature range. The main gel-to-liquid crystalline transition of fully hydrated DPPC is commonly reported at approximately 41 °C; however, transition behaviour can shift or broaden depending on hydration state, sample preparation, and lipid organisation. In low-hydration or complex lipid systems, broader endothermic events at higher temperatures may also be observed [26,27]. As expected, pure cholesterol did not exhibit a distinct transition peak in the examined temperature range, consistent with its role as a membrane fluidity modulator. When incorporated into the lipid matrix, cholesterol can broaden and reduce the enthalpy of DPPC phase transitions. At cholesterol concentrations of 20% *w*/*w* or above, such as the 30% *w*/*w* concentration used in this study, DPPC transition peaks are expected to become less defined, reflecting the ability of cholesterol to alter lipid packing and reduce cooperative phase transitions [28,29,30].

Pure CyA did not show any sharp peak, in agreement with its well-known melting point in the range of 148–151 °C. Regarding the SHN formulations, no distinct peaks were detected, suggesting the successful encapsulation of the drug within the lipid–polymer matrix. However, since the analysed temperature range did not extend to the CyA melting point, this finding should be regarded as indirect evidence rather than direct confirmation of a crystalline-to-amorphous transition of the drug.

### 4.7. Encapsulation Efficiency (EE) and In Vitro Drug Release Profile

Microfluidics is increasingly recognised as a valuable manufacturing approach for drug delivery systems because it can overcome limitations associated with conventional preparation techniques. Among its advantages, microfluidics can improve formulation reproducibility and encapsulation efficiency [5]. CyA was used as a model cyclic peptide with a molecular weight below 2 kDa and, to the best of our knowledge, has not previously been encapsulated in SHNs using microfluidics. Using this approach, a promising encapsulation efficiency of 80 ± 1.765% was achieved in CyA-loaded SHNs, corresponding to approximately 83 μg/mL CyA. This value may also be influenced by the selected lipid composition. Previous studies have shown that DPPC and cholesterol, typically around 30% (*w*/*w*), can influence API encapsulation, particularly for hydrophobic APIs, because cholesterol can increase membrane hydrophobicity and promote drug partitioning within the lipid phase [31]. Considering a typical nebulisation volume of 2–5 mL, the estimated API delivered dose would be in the range of approximately 166–415 μg, although the actual delivered lung dose would require both aerosolisation and deposition studies.

**Figure 11:**
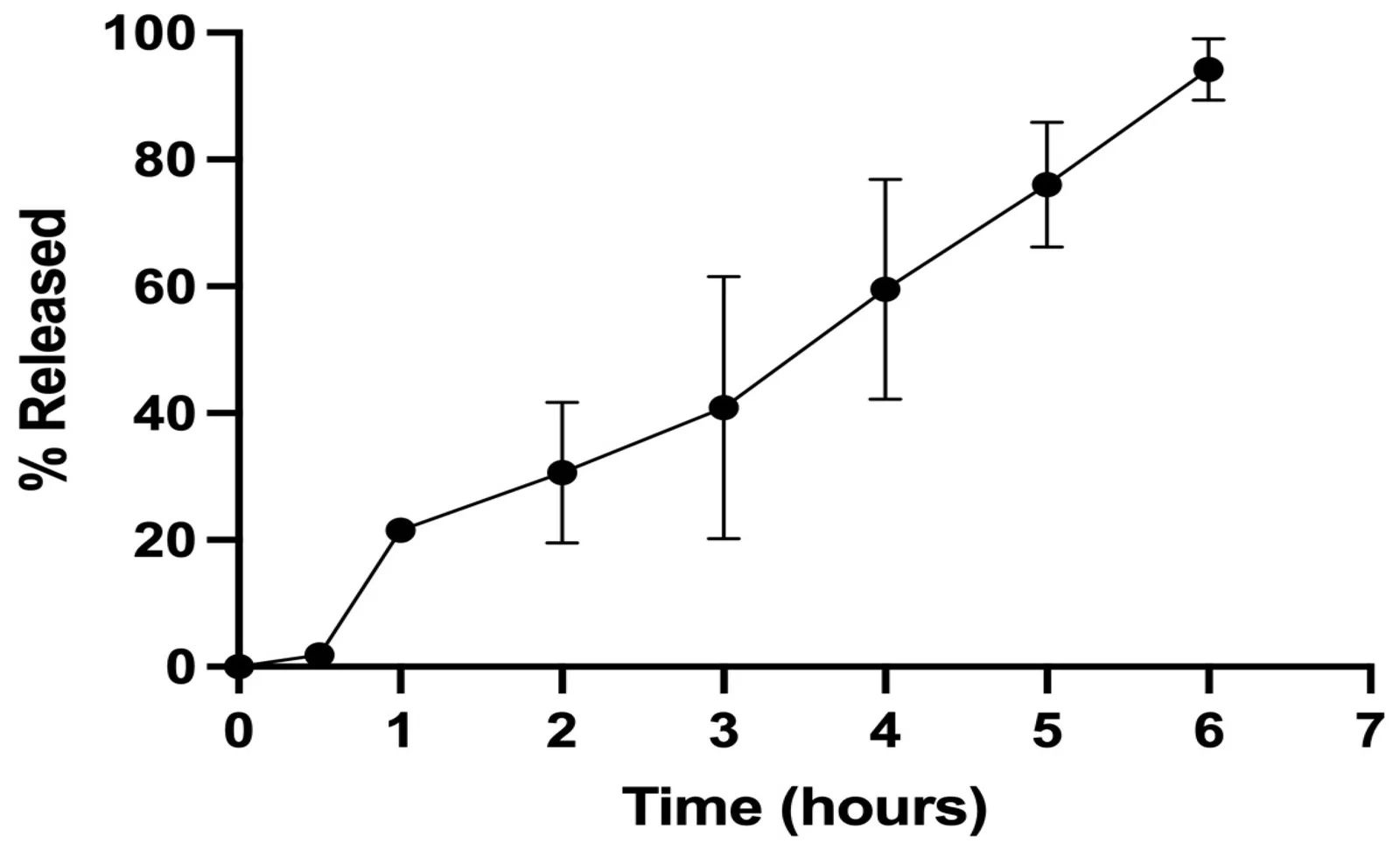
In vitro release profile of CyA from SHNs in PBS as a function of time. The cumulative percentage of CyA released was measured over 6 h under sink conditions.

A 6 h release window was selected as an initial comparative in vitro timeframe to assess the release behaviour of the developed formulation, rather than as a direct prediction of pulmonary dosing. It should be noted that this in vitro release study does not simulate the complex physiological environment of the respiratory tract, including mucociliary clearance, enzymatic degradation, and epithelial absorption, which would significantly influence in vivo release profile and bioavailability. As shown in Figure 11, the release profile showed an initial burst release phase during the first hour, reaching approximately 20%, which may be attributed to diffusion of drug associated with, or located near, the particle surface. This was followed by a more gradual release phase, reaching 94.24 ± 4.84% cumulative release within 6 h. To quantitatively justify this release study, the Korsmeyer–Peppas model was used as the standard kinetic model. The release profile exhibited an R^2^ of 0.983, giving an *n* value of 0.955. In this case, an exponent *n* > 0.89 characterises a Super Case II transport [32], indicating that drug release is primarily controlled by polymer chain relaxation and structural alterations of the formulation [33]. The relatively large standard deviations at intermediate timepoints may reflect variability in diffusion kinetics, particle population, or drug distribution within the formulation.

In general, the release profile indicated controlled and sustained drug release over the investigated period, supporting further development for applications requiring prolonged drug availability over several hours.

## 5. Conclusions, Limitations and Future Perspectives

In this study, SLNs and SHNs were successfully produced using a microfluidic approach. Rapid and controlled mixing in microfluidic nanoprecipitation to limit particle growth and aggregation [34,35,36] was shown to be highly effective in generating nanoparticles with well-controlled, narrow size distributions and good batch-to-batch reproducibility, confirming its suitability for scalable nanomedicines manufacturing [36].

SHNs loaded with Cyclosporine A exhibited favourable physicochemical properties, with particle sizes below 200 nm, PDI values within acceptable limits, and a positive ζ potential conferred by the chitosan coating. DLS and TEM analyses supported the formation of nanoscale hybrid nanoparticles, while TEM images showed a morphology consistent with the proposed lipid–chitosan architecture. FTIR, TGA, and DSC supported CyA incorporation and compatibility with the excipients, although these techniques should be interpreted as complementary rather than definitive evidence of molecular arrangement or core–shell confirmation.

SHNs retained their physicochemical integrity for 28 days under all tested storage conditions and showed an encapsulation efficiency of approximately 80%, a high value for a hydrophobic cyclic peptide such as CyA. This performance may be supported by the DPPC- and cholesterol-rich lipid composition, which can enhance membrane hydrophobicity and drug partitioning within the lipid core [37]. The in vitro release profile showed an initial burst release of approximately 20% within the first hour, followed by sustained release reaching 94.24 ± 4.84% cumulative release within 6 h.

Overall, the combination of controlled particle size, positive surface charge, high encapsulation efficiency, and sustained release profile highlights the potential of this hybrid system as a candidate carrier for hydrophobic cyclic peptides. However, the obtained data should be considered preliminary with respect to pulmonary delivery performance.

While the physicochemical characterisation, stability, and in vitro release behaviour of SHNs were investigated, several aspects relevant to their translational potential as pulmonary delivery systems remain to be addressed and were beyond the scope of the present work. Future studies should evaluate aerosolisation performance, aerodynamic deposition, nebulisation stability, mucus interaction, cytocompatibility, and in vivo behaviour to establish the translational potential of microfluidic-produced SHNs as a pulmonary delivery platform for CyA and other hydrophobic cyclic peptides.

## Figures and Tables

**Table 1:** Parameters selected and used for empty SLN production.

Name	DPPC	Chol	TFR (mL/min)	FRR (Organic/Aqueous)
F1	70	30	0.5	1:2
F2	70	30	1	1:2
F3	70	30	2	1:2
F4	70	30	4	1:2

**Table 2:** Polydispersity index (PDI) and ζ potential of lipid nanoparticle formulations F1–F4. Data are reported as means ± standard deviations (SDs).

Name	PDI	ζ Potential
F1	0.20 ± 0.02	−11.6 ± 0.854
F2	0.19 ± 0.08	−4.04 ± 1.23
F3	0.095 ± 0.041	−2.75 ± 2.00
F4	0.057 ± 0.043	−7.74 ± 1.93

**Table 3:** Particle size, PDI, and ζ potential of loaded SHNs. Data are reported as means ± standard deviations (SDs). All size measurements were taken on day 0 of formulation, and samples were analysed in triplicate.

Name	TFR/FRR	Diameter (nm)	SDS	PDI	SDS	ζ Potential (mV)	SDS
SHNs_1	TFR 2/1:2	168.72	±4.75	0.22	±0.022	+18.97	±1.29
SHNs_2	TFR 2/1:2	145.86	±3.95	0.19	±0.020	+11.61	±1.85
SHNs_3	TFR 2/1:2	167.03	±10.81	0.21	±0.036	+ 17.81	±0.32

**Table 4:** PDI and ζ potential values were monitored on SHNs over a 28-day period, using 4/25/37 °C as storage conditions.

T (°C)	PDI	SDS	ζ Potential (mV)	SDS
4 °C	0.214	±0.018	+14.24	±2.02
25 °C	0.191	±0.037	+15.2	±1.19
37 °C	0.199	±0.022	+13.32	±1.57

## Data Availability

Data available on request due to restrictions, e.g., privacy or ethical.

## Abbreviations

The following abbreviations are used in this manuscript:

SLNs	Solid Lipid Nanoparticles
SHNs	Solid Hybrid Nanoparticles
CyA	Cyclosporine A
DPPC	1,2-Dipalmitoyl-sn-glycero-3-phosphocholine
DLS	Dynamic Light Scattering
TEM	Transmission Electron Microscopy
TGA	Thermogravimetric Analysis
DSC	Differential Scanning Calorimetry
COPD	Chronic Obstructive Pulmonary Disease
NPs	Nanoparticles
MF	Microfluidics
DDSs	Drug Delivery Systems
CAD	Computer-Aided Design
TFRs	Total Flow Rates
FRRs	Flow Rate Ratios
PDI	Polydispersity Index
PBS	Phosphate-Buffered Saline
DI	Deionised water
DF	Dilution Factor
PS	Pulmonary Surfactant
